# Intronization Signatures in Coding Exons Reveal the Evolutionary Fluidity of Eukaryotic Gene Architecture

**DOI:** 10.3390/microorganisms10101901

**Published:** 2022-09-25

**Authors:** Judith Ryll, Rebecca Rothering, Francesco Catania

**Affiliations:** 1 Institute for Evolution and Biodiversity, University of Münster, Hüfferstrasse 1, 48149 Münster, Germany; 2 Institute of Environmental Radioactivity, Fukushima University, Fukushima 960-1248, Kanayagawa, Japan

**Keywords:** gene architecture, intron, exon, RNA splicing, alternative splicing, purifying selection, intronization, exonization, gene expression

## Abstract

The conventionally clear distinction between exons and introns in eukaryotic genes is actually blurred. To illustrate this point, consider sequences that are retained in mature mRNAs about 50% of the time: how should they be classified? Moreover, although it is clear that RNA splicing influences gene expression levels and is an integral part of interdependent cellular networks, introns continue to be regarded as accidental insertions; exogenous sequences whose evolutionary origin is independent of mRNA-associated processes and somewhat still elusive. Here, we present evidence that aids to resolve this disconnect between conventional views about introns and current knowledge about the role of RNA splicing in the eukaryotic cell. We first show that coding sequences flanked by cryptic splice sites are negatively selected on a genome-wide scale in *Paramecium*. Then, we exploit selection intensity to infer splicing-related evolutionary dynamics. Our analyses suggest that intron gain begins as a splicing error, involves a transient phase of alternative splicing, and is preferentially completed at the 5’ end of genes, which through intron gain can become highly expressed. We conclude that relaxed selective constraints may promote biological complexity in *Paramecium* and that the relationship between exons and introns is fluid on an evolutionary scale.

## 1. Introduction

Two studies published in 1977 [1,2] have shaped our current view of genes. In the words of Gilbert, “coding sequences on DNA […] are not contiguous but are interrupted by ‘silent’ DNA” [3]. These silent DNA sequences have since been called spliceosomal introns (hereinafter introns). Their flanking sequences were dubbed exons. The separation of intragenic sequences into introns and exons is deeply entrenched in contemporary biological thinking [4,5]. Exons participate in protein synthesis, whereas introns must be removed by the spliceosome, in a process known as RNA splicing, to generate functional proteins.

RNA splicing is commonly viewed as the nuclear process that single-handedly removes introns from precursor mRNAs and joins exons together. This view of RNA splicing as an intron-removing operation has become too narrow, however. Because of its intimate connections with other mRNA-associated processes [6], RNA splicing can influence other cellular processes’ efficiency and outcome. In one example, the U1 small nuclear ribonucleoprotein (snRNP), which triggers splicing upon binding to 5’ splice sites (5’ss), also exerts distance-dependent inhibitory effects on polyadenylation factors [7,8]. These effects have been dubbed telescripting and can prevent unwanted premature polyadenylation in the internal regions of nascent transcripts as well as modulate the efficiency of mRNA processing at the transcript 3’ end [9]. In turn, polyadenylation factors that bind to nascent transcripts can affect the splicing efficiency of encompassing or proximal introns [10,11].

Furthermore, the product of RNA splicing is not limited to intron removal. Across most species, RNA splicing promotes the exclusion/inclusion of both coding and non-coding mRNA sequences in multifarious and alternative combinations [12]. This *modus operandi* of RNA splicing generates considerable biological variation, some with selectively neutral consequences [13,14], some with functional roles [15], and some with detrimental effects on health [16]. Importantly, this *modus operandi* of RNA splicing blurs the original distinction between exons and introns. For example, what should one call sequences that are retained in mature mRNAs about 50% of the time? While exposing the limitations of the exon and intron labeling, the very existence of alternative RNA splicing suggests that exonic and intronic sequences may be less distinct from each other than conventionally thought [17,18,19,20]. Thinking of RNA splicing as more than an intron-removing process can help generate models of gene architectural and regulatory dynamics that integrate the evolution of gene structure with the molecular mechanisms that underlay splice site selection and intron turnover [19]. Reasoning beyond the intron-exon dichotomy could also help solve one of the biggest mysteries in biology over the past four decades: Where do introns come from?

Recently, a DNA transposon mechanism was proposed to generate introns on a genome-wide scale [21]. Upon insertion of non-autonomous DNA transposons, the spliceosome of the algae *Micromonas* and *Aureococcus* recognizes a splice site at one end of the transposons and a splice site that is co-opted from the adjacent gene sequence. The authors of this study plausibly concluded that DNA transposons and sequence co-option may account for episodes of rapid, extensive intron gain during eukaryotic evolution [21]. In a more recent example of a genome-wide mechanism of intron gain, Talkish and colleagues [22] reported >150 splicing episodes in non-intronic intragenic regions after artificially enhancing the activity of the spliceosome in budding yeast. These new splicing events require no transposition. However, like the introns described by Huff and colleagues [21], they rely on the co-option of latent or cryptic splice sites. Altogether, these and other observations [23,24] suggest that the co-option of pre-existing cryptic splice sites is key to the emergence of introns. 

This latter idea is at the core of the Intronization Model (IM) [17,19,20]. Under IM, the emergence of introns from coding sequences can be divided into three temporal phases. First, splicing factors recognize consecutive cryptic splice sites along nascent transcripts and occasionally trigger splicing. Sufficiently large coding sequences that are positioned anywhere along the transcript may undergo fortuitous splicing. However, coding sequences that are short and have a size that is a multiple of 3 (3*n*) are most likely to ultimately convert into spliceosomal introns. RNA splicing of short and 3*n* coding sequences is expected to least impact protein function. Moreover, the Cap-Binding-Complex (CBC) locally enhances the recruitment of U1 [25,26,27], a key splicing-factor that triggers spliceosome assembly in the absence of ATP. Thus, IM also maintains that fortuitous splicing occurs at the pre-mRNA 5’ end more often than at its 3’ end. This latter aspect can have phenotypic consequences because efficient splicing at the pre-mRNA 5’ end can enhance gene expression [28,29,30,31]. 

Second, as fortuitous splicing generates spliced and un-spliced isoforms at a given locus, this state of alternative splicing may persist, particularly in conditions of relaxed selective constraints. Under IM, alternative splicing is a key step in the transition between exonic and intronic sequences [17]. In the same way, alternative splicing is a key step in the transition between intronic and exonic regions in the reverse process of exonization [18,32]. 

Third and last, the short 3*n* coding sequences that undergo the intronization path and are alternatively spliced acquire a premature termination codon (PTC). Consequently, the un-spliced isoform is degraded via the nonsense-mediated decay (NMD) system, whereas the spliced (PTC-free) sister isoform is spared. This means that the efficient degradation of the un-spliced isoform promotes the establishment of the spliced sequence. Because NMD-mediated degradation is generally less efficient when PTCs reside toward the transcript 3’ end [33,34,35], splicing at the pre-mRNA 5’ end is expected to become constitutively more rapidly compared to splicing at the pre-mRNA 3’ end. Once a new constitutively spliced intron is gained, its size may increase at the expense of flanking exon sequences [20]. 

Numerous episodes of intronization have been reported across eukaryotes [36,37,38,39,40,41,42,43,44,45]. However, the extent to which intronization accounts for intron gain at the genome scale in the absence of transposition events, or without artificially enhancing splicing activity remains unknown. Here, we address this question using the free-living ciliate *Paramecium tetraurelia* (*Paramecium* henceforth). 

## 2. Materials and Methods

### 2.1. Paramecium Strain and Datasets

The macronuclear reference genome (v1.0) [46,47] and annotation (v2.0) of *Paramecium tetraurelia* strain 51 [48] (publicly available through parameciumDB [49]) were used to extract information relative to annotated introns and coding exons. Published gene expression data from *P. tetraurelia* strain 51 (vegetative phase, [48]) were used to group genes in quartile-based classes after log2-transformation. Last, we used RNAseq reads from two control samples of vegetatively growing cells (*Paramecium* strain 51; tK2 and tK3, accession numbers ERR1676709 and ERR1676710, respectively) from [50] to investigate variation in the splicing profile of annotated introns and collect novel (i.e., non-annotated) splice junctions.

### 2.2. Transcriptomic Data Processing

Sequence reads were adapter-trimmed with Atropos (v.1.1.5) [51] using the insert-match algorithm, default values for the error rates, and ensuring a minimum length of 35 bp after trimming. BBMerge (v.37.25) [52] was used with the “ecco mix” option for overlap-based error correction. Preprocessed reads were mapped to the reference *P. tetraurelia* genome with STAR (v.2.5.3a) [53] in the two-pass mode, setting the minimal and maximal intron lengths to 10 and 500 nt, respectively, and with soft-clipping of reads disabled. Further, the maximum mismatch rate was set dynamically by specifying --outFilterMismatchNoverLmax 0.02, the scoring bonus for spliced alignments disabled with --sjdbScore 0, sensitivity in the seed search step increased by --seedSearchStartLmax 25 and reads with a single best-scoring alignment considered uniquely mapping via --outFilterMultimapScoreRange 0.

### 2.3. Extraction of GT|AG Coding Sequences

We used *P. tetraurelia*’s coding exome to extract all the possible segments bordered by GT and AG (GT|AG) along sense (N = 3,420,273) and antisense (N = 2,475,164) DNA strands. We focused on segments that are 15 to 40 nt long; a size distribution that comprises >99% of annotated introns in *P. tetraurelia*. For each of the surveyed size classes, we estimated the degree of DNA strand asymmetry (DSA, see below). Additionally, we matched each of the GT|AG coding sequences isolated from the sense strand with novel (i.e., non-annotated) splice junctions that the program STAR detected in the two surveyed replicate transcriptomes.

### 2.4. DNA Strand Asymmetry (DSA)

According to a neutral model of evolution, mono- and oligonucleotides and their reverse complements occur in equal amounts in the opposite strands. If so, then the degree of DSA allows conclusions to be drawn on the selective pressure to which a sequence motif is subjected. Negative DSA values for a surveyed sequence motif reveal under-representation in the sense strand and are therefore indicative of negative selection. We estimated the DSA degree for consecutive GT and AG dinucleotides at a distance ranging between 15 and 40 nt using Equation (1), where S is the asymmetry score and Ns and Na are the counts of consecutive GT and AG dinucleotides on the sense and antisense DNA strand, respectively:

S = (Ns − Na)/(Ns + Na) (1)

We further calculated the DSA degree for GTA|TAG coding sequences residing in the first, internal, and last exons as well as highly and weakly expressed genes. To create a distribution that would allow us to statistically test for differences in DSA between different exon positions and expression levels, we randomly drew 500 genes, summed Ns and Na, and estimated the DSA using Equation (1) for each of these groups. These analyses were repeated 1000 times, yielding 1000 DSA scores for each first, internal, and last exons and high and weak expression levels. Additionally, we examined the DSA of optimal splicing signals in the exonic sequences flanking true introns. More specifically, we calculated the DSA for the GTA or TAG trinucleotide in the 15 nt upstream of the constitutive 5′ss or downstream of the constitutive 3’ splice site (3’ss), respectively.

### 2.5. Quantification of Splicing Events

We examined splicing events following a similar approach as in [50]. Specifically, we used in-house Python scripts to collect uniquely mapped reads that support retention of annotated introns and GT|AG coding sequences with splicing evidence. In case both reads of a pair overlapped and supported retention of the same intron/GT|AG coding sequence, we counted a single retention event to align our counting procedure with the one for spliced reads reported by STAR. Reads were only considered to support retention when they covered the entire length of the annotated intron. Additionally, we grouped all splice junctions reported by STAR based on their genomic positions to identify alternative splicing variants, i.e., junctions sharing either the 5’ss or the 3’ss. For each intron/GT|AG coding sequence, we determined the level of splicing, retention, and alternative splice site usage as the proportion of reads from the respective category out of all analyzed reads related to the intron/GT|AG coding sequence under focus. 

## 3. Results

### 3.1. Intron Properties and Optimal Splice Sites in the Ciliate Paramecium

*Paramecium* genes contain a relatively high intron density (2.9 introns, on average) and large exons (the average [median] size of CDS exons in intron-containing genes is 354 bp [194 bp]) [46,48]. This ciliate’s introns are extremely short: they average ~25 bp and ~95% of them fall in a 21–30 bp size range. More than two thirds (69%) of its annotated 94,711 CDS introns (the miniscule UTRs with 283 introns are not considered in this study) contain GTA and TAG at their 5’ and 3’ ends, respectively. Note that TAG and TAA, both stop codons in the standard genetic code, in *Paramecium* are instead reassigned to encode glutamine (making TGA the only stop codon in this species). At least three pieces of evidence suggest that GTA and TAG are optimal splice sites (i.e., preferential targets of the spliceosome) in *Paramecium*. First, GTA|TAG configurations, where “|” refers to the encompassed sequence, are twice more common within, rather than outside, the prevalent 21–30 bp size range. Second, our re-analysis of previously published transcriptomic data [50] shows that GTA|TAG introns experience less alternative/erroneous splicing than non-GTA|TAG introns (average of two replicates; alternative splice site usage: 9% vs. 13%; intron retention: 50% vs. 58%; proportion tests, all *p*-values < 2.2 × 10^−16^). Third and lastly, GTA|TAG introns are most frequent in highly expressed genes (Table 1), where the pressure to reduce the negative effects of erroneous splicing is presumably higher, on average, compared to genes with reduced expression. Against this background, we reasoned that if introns originate from the fortuitous splicing of coding sequences, then GTA|TAG coding regions should be the most likely source of new introns in *Paramecium*. The next step is to test the hypothesis that GTA|TAG coding regions have the potential to turn into introns.

### 3.2. Intronization Signature in the Paramecium Genome

Annotated introns in *Paramecium* are generally shared between conspecific strains and closely related species [49,54]. Thus, if intronic sequences in *Paramecium* originate by means of intronization of coding sequences, then intronization must have been particularly active in ancestral genes but is somehow attenuated in modern genes. Through the lens of IM, this attenuation may reflect a relative dearth of coding sequences flanked by 5’-GTA and TAG-3’. To test this, we extracted all the annotated exons in *Paramecium* [48] and determined the number of consecutive GT and AG dinucleotides that are 15 to 40 nt apart from one another on the DNA sense and antisense strands. With these counts in hand, we computed the degree of DNA strand asymmetry (DSA) [55,56] *per* size class. 

**Figure 1:**
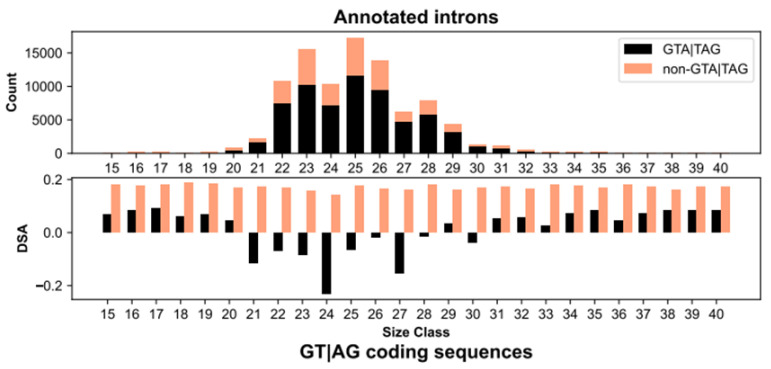
Intronization signature in the *Paramecium* genome. Top: Size distribution of annotated introns. The 94,711 CDS introns in *Paramecium* are extremely short, very narrowly distributed, and depleted in sizes that are a multiple of 3. Configurations with GTA and TAG at the 5’ and 3’ splice sites, respectively, (black) are more prevalent than all non-GTA|TAG configurations (light salmon) taken together. Bottom: DNA strand asymmetry (DSA) values of GT|AG coding sequences. GTA|TAG (black), but not non-GTA|TAG (light salmon) exonic segments, are counter-selected (as indicated by their negative DSA values) in the size range where annotated introns are most common. This counter-selection is especially pronounced when their size is a multiple of 3. Only introns between 15 and 40 nt, which comprise >99% of them, are shown.

**Figure 2:**
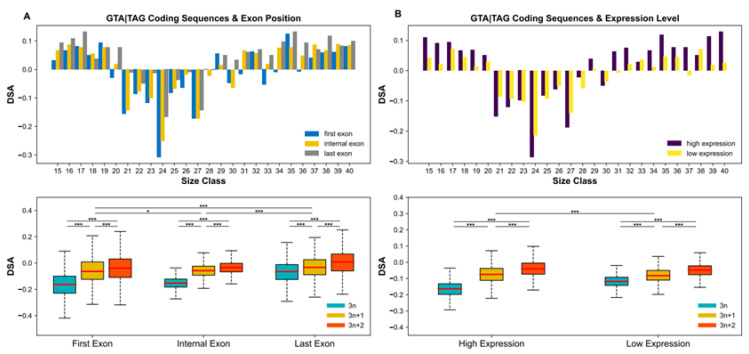
DNA strand asymmetry (DSA) variation along genes and between levels of gene expression. (**A**) Intronization signature in coding exons according to their position along genes. Top: DSA values for first exons (blue), internal exons (yellow), and last exons (grey). Bottom: Within the range of prevalent intron sizes (21–30 bp), 3*n* (turquoise) GTA|TAG coding sequences have lower DSA values than their 3*n* + 1 (mustard yellow) and 3*n* + 2 (red) counterparts, irrespective of the positional class of their exon. (**B**) Intronization signature in exons according to the expression level of their gene. Top: DSA values of GTA|TAG coding sequences in highly (dark violet) and weakly (yellow) expressed genes. Bottom: In both highly and weakly expressed genes, 3*n* (turquoise) GTA|TAG coding sequences in the prevalent intron size range (21–30 bp) display more negative DSA values than 3*n* + 1 (mustard yellow) and 3*n* + 2 (red) ones. *** *p*-value < 0.0001; * *p*-value < 0.05 (*t*-test; Bonferroni corrected).

The resulting distribution of DSA values reveals a striking nonrandom pattern (Figure 1). Against a background of positive DSA estimates, which presumably reflect the contribution of GT and AG dinucleotides to amino acid sequences, coding sequences flanked by 5’-GTA and TAG-3’ (i.e., GTA|TAG) exhibit negative DSA values, and are indicative of a deficit due to purifying selection. The range of these DSA values mirrors the frequency distribution of annotated introns (Figure 1), and their magnitude exhibits biases along genes and between gene expression classes (Figure 2). Namely, there is a trend towards more negative DSA values in first coding exons compared to internal (t-test; Bonferroni corrected *p* = 0.0169) and last exons (t-test; Bonferroni corrected *p* < 2.2 × 10^−16^) (Figure 2A), and in highly expressed genes compared to weakly expressed genes (t-test; Bonferroni corrected *p* = 6.9 × 10^−11^) (Figure 2B). In both the exon position and the gene expression datasets, GTA|TAG coding sequences within the range of prevalent intron sizes (21–30 bp) and with a length divisible by 3 (3*n*), display the most pronounced negative DSA values (t-test; all Bonferroni corrected *p*-values < 0.0001; Figure 2A,B). These findings fit well with the IM prediction that 3*n* coding sequences at the gene 5’ end are the most likely to convert into spliceosomal introns over evolutionary time.

### 3.3. The First Two Phases of the Intronization Process

**Figure 3:**
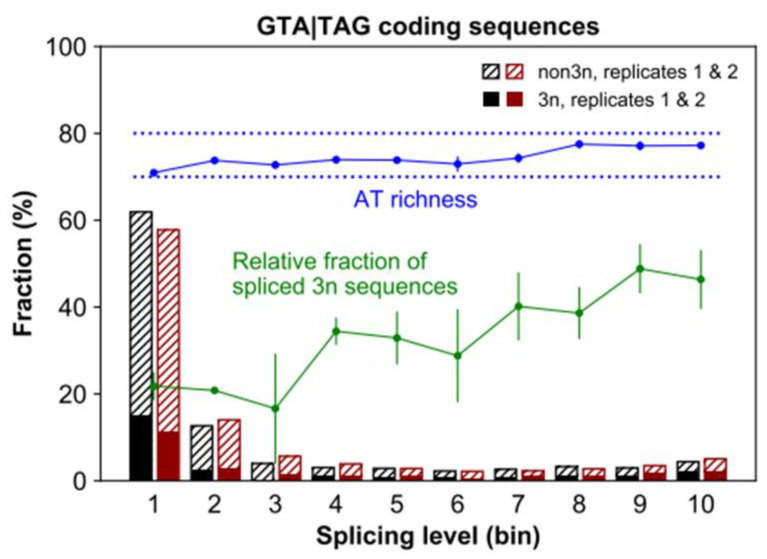
Splicing levels of GTA|TAG coding sequences. Stacked bar heights represent the percentage of spliced sequences in each splicing level class (one of ten equal-width bins, e.g., bins 1 and 2 represent the splicing level intervals (0, 0.1] and (0.1, 0.2], respectively). Only loci covered by at least ten reads are considered. Solid and dashed bar parts (colored in black or red for the two surveyed replicates) correspond to 3*n* and non-3*n* spliced sequences, respectively. Further, the green and blue line plots show the relative fraction of 3*n* sequences and the AT content of the spliced sequences per splicing level class, respectively (mean +/− standard deviation of the two replicates in both cases).

The footprint of negative selection that we describe above can be interpreted as an “*intronization signature*”. Specifically, GTA|TAG coding sequences are largely counter-selected because their conversion to introns would be deleterious in the prevalent modern environment. If so, then one may expect that GTA|TAG coding sequences in *Paramecium* are particularly susceptible to splicing. Furthermore, *Paramecium*’s spliceosome should recognize/remove GTA|TAG coding sequences to an extent that is sufficiently large to induce a selective response. We tested these predictions leveraging recently published transcriptomic data obtained from the same *Paramecium* strain under study [50]. We find that 8320 sequences annotated as coding exons do experience splicing in at least one of two studied replicates (Rep1 and Rep2) (Figure 3). Most of these non-annotated splice junctions (86%, N = 7137) display GTA at their 5’ end. About 37% of them are simultaneously flanked by 5’-GTA and TAG-3’, a significant excess compared to the non-GTA|TAG counterpart (1.4% vs. 0.2% relative to the total number of cryptic splice sites; proportion test, *p* < 2.2 × 10^−16^).

Additionally, although most spliced GTA|TAG coding sequences experience low-level splicing (<10%, Figure 3), the fraction of 3*n* sequences increases with increasing splicing level (Rep1, Rep2, Pearson’s *r* > 0.81, *p* < 0.005; Figure 3). Similarly, there is a positive coupling between splicing level and AT content (Rep1, Rep2, Pearson’s *r* > 0.81, *p* < 0.005; Figure 3), which ranges between ~70% and ~80%, i.e., the AT-richness typical of *Paramecium* coding exons and introns, respectively [48]. Thus, besides experiencing the highest level of purifying selection (Figure 1 and Figure 2), 3*n* GTA|TAG coding sequences, when spliced, may reach considerable levels of excision. 

### 3.4. Spliced GTA|TAG Coding Sequences Are Unevenly Distributed along the Genes and Genome of Paramecium

We then examined the splicing level of GTA|TAG coding sequences as a function of gene expression level and intragenic location. 

**Figure 4:**
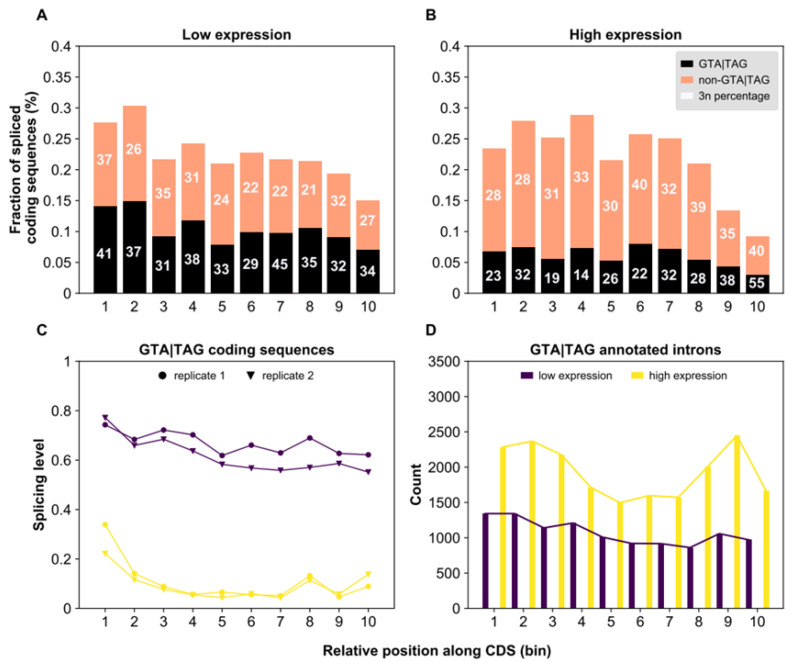
Splicing-related features of spliced coding sequences and annotated introns along genes with different expression levels in *Paramecium*. For both weakly (**A**) and highly (**B**) expressed genes, CDS was divided into ten bins of equal width and the fraction of coding sequences with evidence of splicing (in at least one of the two surveyed replicates) out of all spliced and un-spliced GT|AG coding sequences in each bin was determined. The numbers within the bars correspond to the proportion (expressed in percentage) of coding sequences whose length is a multiple of 3. Irrespective of the gene expression level, the fraction of spliced coding sequences is not uniformly distributed along genes (low expression: χ^2^ = 48.42, df = 9, *p* = 2.13 × 10^−7^; high expression: χ^2^ = 122.57, df = 9, *p* < 2.2 × 10^−16^). The relative fraction of spliced GTA|TAG coding sequences (black portion of bars) is larger in weakly (**A**) than in highly expressed genes (**B**) (46% vs. 27%, respectively; proportion test, *p* < 2.2 × 10^−16^). (**C**) Average splicing levels of spliced GTA|TAG coding sequences in each bin along the CDS are depicted for weakly (dark violet) and highly (yellow) expressed genes. Both the gene expression level and the relative position along the CDS vary with the splicing level of GTA|TAG coding sequences. When comparing (**C**) with (**D**), the spatial distribution of annotated GTA|TAG introns resembles the spatial distribution of spliced GTA|TAG coding sequences. In contrast, the relative excess of spliced GTA|TAG coding sequences in weakly expressed genes is inconsistent with the relative deficit of annotated introns in weakly expressed genes.

We found that the fraction of spliced GTA|TAG coding sequences is higher in weakly expressed genes compared to highly expressed genes (46% vs. 27%, respectively; proportion test, *p* < 2.2 × 10^−16^; Figure 4A,B). Additionally, GTA|TAG coding sequences also show the highest levels of splicing in weakly expressed genes (Figure 4C). In contrast to spliced GTA|TAG coding sequences, GTA|TAG introns preferentially occupy highly expressed genes (Figure 4D). Through the lens of IM, these observations suggest that coding sequences that are most often spliced in weakly expressed genes are more likely to reach an intronic state in highly expressed genes (more below).

Our analyses also revealed that spliced GTA|TAG coding sequences are relatively infrequent at the gene 3’ end (Figure 4A,B) but tend to populate the 5’ terminal region (weakly expressed genes: Pearson’s *r* = −0.763, Bonferroni corrected *p* = 0.020; highly expressed genes: Pearson’s *r* = −0.739, Bonferroni corrected *p* = 0.029). Additionally, *Paramecium*’s internal exons contain a deficit (1.1%) of spliced GTA|TAG sequences compared to first and last exons (2.1% and 1.3%, respectively; proportion test, *p* < 2.2 × 10^−16^ and *p* = 0.0029, respectively; genes with >2 coding exons) (Figure 4C). Finally, spliced internal coding sequences are flanked by 5’-GTA and TAG-3’ less often than spliced terminal coding sequences (34.9% vs. 39.2% and 39.2% for internal, first, and last exons, respectively; genes with >2 coding exons; proportion test, *p =* 0.0095 and *p =* 0.012, respectively). Under IM, these observations imply that if intronization of coding sequences truly occurs, then it must be disfavored in the internal regions of many modern *Paramecium* genes.

**Figure 5:**
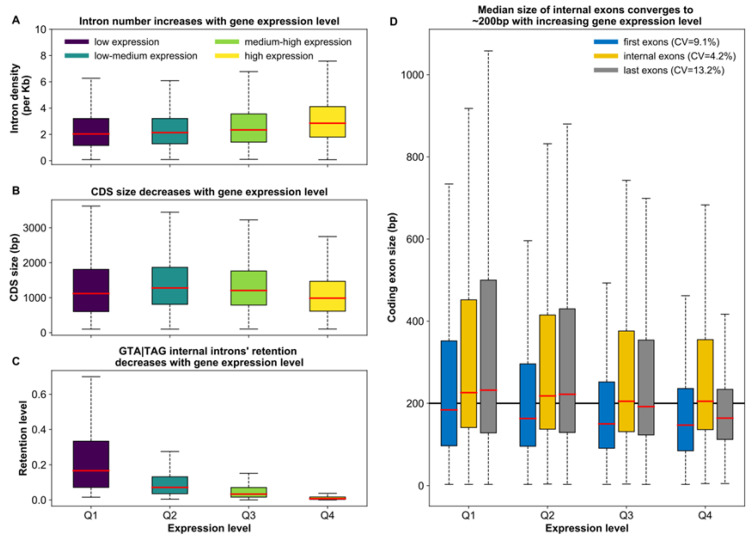
Relationships between expression and architectural properties of genes in *Paramecium*. (**A**) Intron density increases with gene expression level. The number of introns per kilobase of coding sequence, i.e., the intron density, was determined for genes of different expression level quartiles (Q1 to Q4). Median intron density (red lines in boxes) is smallest in weakly expressed genes (dark violet, Q1) and greatest in highly expressed genes (yellow, Q4) (2.0 vs. 2.8 introns per kb of coding sequence in weakly and highly expressed genes, respectively; Wilcoxon rank sum test, *p* < 2.2 × 10^−16^). (**B**) CDS length in *Paramecium* decreases with the expression level of the gene. In highly expressed genes (yellow, Q4), median CDS length (red line in box) is shorter and CDS length varies less than in genes of other expression level quartiles (987 vs. 1119 bp in highly and weakly expressed genes, respectively; Wilcoxon rank sum test, *p* < 2.2 × 10^−16^). (**C**) The retention level of internal GTA|TAG introns decreases with gene expression level. Introns with non-zero retention in at least one of the two studied replicates were grouped by the expression level of their gene. Boxes show the average retention levels of the two replicates for each of these introns. In weakly expressed genes (dark violet, Q1), retention levels vary considerably more, and median retention (red line in boxes) is higher than in genes of the other expression quartiles. (**D**) Length variation of *Paramecium* exons according to their position (first, internal, or last; colored in blue, yellow and grey, respectively) and expression level quartile of their gene (x-axis, Q1 to Q4). Only genes with at least 3 exons are considered. Exons tend to be shortest and exon length most narrowly distributed in highly expressed genes (Q4), irrespective of the exon’s position in the gene. Across different expression levels, the median length of internal exons varies less than it does in first and last exons, as indicated by the coefficient of variation around the medians in the legend. With increasing expression level, the median size (red line inside boxes) of internal exons converges to ~200 bp (marked by the black horizontal line)—which is also the distance between true introns and potentially cryptic introns, around which the latter experience the highest levels of splicing. (**A**–**D**) Outliers have been omitted for visualization purposes.

**Figure 6:**
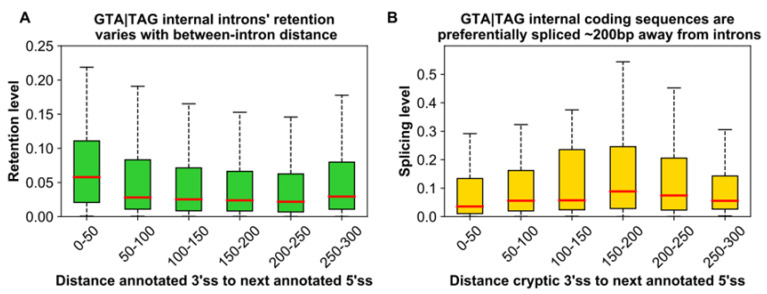
Splicing-related features of GTA|TAG annotated introns and spliced coding sequences in the internal regions of *Paramecium* genes (**A**) The retention level of annotated internal GTA|TAG introns varies with between-intron distance. Average retention levels were determined for introns with non-zero retention in at least one of the two studied replicates, after grouping introns according to the distance between their 3′ss and the 5′ss of the next downstream intron. Introns that are located very closely to one another (leftmost box) have higher retention levels than introns located ~200 bp apart. (**B**) The splicing level of internal GTA|TAG coding sequences varies with the distance to the next downstream intron. GTA|TAG internal coding sequences with evidence of splicing in at least one of the two surveyed replicates were classified according to the distance between their 3′ end and the 5′ss of the following annotated intron. The average splicing level of the two replicates was then determined. Median splicing level (red line in boxes) is highest when this distance is ~200 bp. (**A**,**B**) Outliers have been omitted for visualization purposes.

Investigating this further, we found that gene expression level is *positively* correlated with intron density (Pearson’s *r* = 0.151, *p* < 2.2 × 10^−16^; Figure 5A), but *negatively* correlated with the length of the CDS (Pearson’s *r* = −0.099, *p* < 2.2 × 10^−16^; Figure 5B). Thus, highly expressed genes in *Paramecium* show a particularly high intron density. The internal introns of these highly expressed genes show peculiar features. Not only does their level of accurate splicing increase progressively as we move from weakly to highly expressed genes (Figure 5C), but their reciprocal median distance converges toward ~200 bp (Figure 5D). This convergence hints at an association between inter-intron distance and splicing efficiency, which aligns well with two other observed trends. First, annotated introns that are spaced less than ~200 bp apart are relatively more likely to experience retention (Figure 6A). Second, the splicing level of GTA|TAG internal coding sequences is the highest at around 200 bp from the next 5’ss of an annotated intron (Figure 6B).

Taken together, these results indicate that the probability of splicing along coding sequences may depend, *inter alia*, on the size of the intervening exon. Thus, spatial constraints might contribute to hindering intron colonization in the inner regions of modern *Paramecium* genes. 

### 3.5. The Final Phase of the Intronization Process

**Figure 7:**
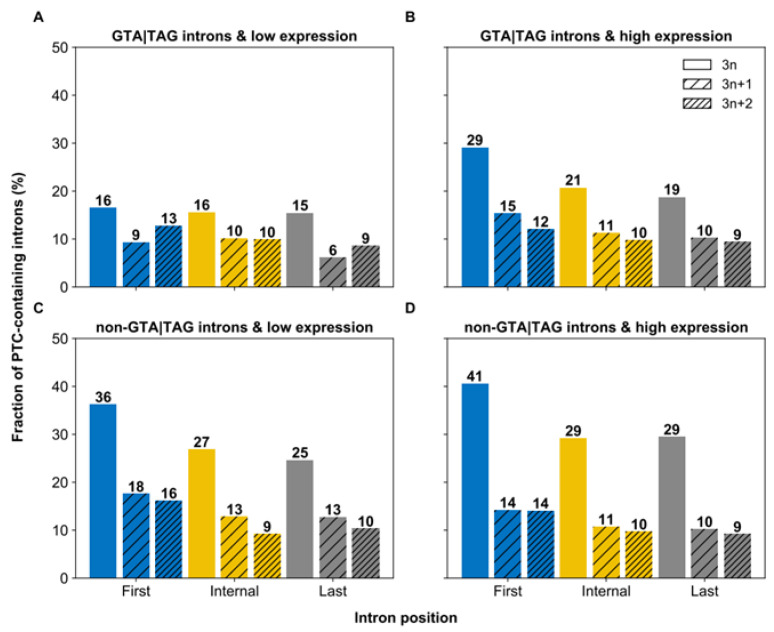
Spatial distribution of PTC-containing introns in relation to gene expression levels. Fraction of PTC-containing GTA|TAG (**A**,**B**) and non-GTA|TAG (**C**,**D**) introns in different positional (first, internal, and last in genes with >2 introns) and length (3*n*, 3*n* + 1 or 3*n* + 2) classes for genes with low (**A**,**C**) and high (**B**,**D**) expression levels. Introns that belong to the 3*n* length class are more often PTC-containing than those of the other two length classes, irrespective of their position, their splicing signals, or the expression level of their gene, and 3*n* and PTC-containing introns occur more frequently at the gene 5’ end of highly expressed genes than weakly expressed genes, when they have strong (**B** vs. **A**) but not weak (**D** vs. **C**) splicing signals.

According to IM, the journey of spliced 3*n* coding sequences toward constitutive splicing reaches completion when these sequences acquire PTCs. This predicts that annotated 3*n* introns in *Paramecium* may be preferentially enriched with PTCs. Indeed, *Paramecium*’s 3*n* introns are ~twice more likely to contain PTCs than 3*n* + 1 and 3*n* + 2 introns (22.6% vs. 11.7% and 11.3%, respectively; proportion test, *p* < 2.2 × 10^−16^). This result aligns with previous (partly EST-based) observations in *Paramecium* [17,57], other ciliates [58], and five additional non-ciliate eukaryotes including *H. sapiens* [57]. Furthermore, although PTC^+^ 3*n* introns preferentially occur at the gene 5’ end, regardless of their splice site strength (GTA|TAG: 24% vs. 19% and 17%; non-GTA|TAG: 34% vs. 27% and 26% for first, internal and last introns, respectively; proportion test, *p* ≤ 7.77 × 10^−5^), this positional bias affects solely highly expressed genes in the case of GTA|TAG introns (proportion test, *p* = 7.24 × 10^−7^ and *p* = 0.9088 for highly and weakly expressed genes, respectively) (Figure 7). These observations are in line with the IM proposition that 3*n* coding sequences, especially those that reside at the gene 5’ end, are the most likely to take the intronization path and complete it. They also indicate that highly expressed genes in *Paramecium* preferentially accrue introns, consistent with these genes’ DSA profile (Figure 2) and elevated intron richness (Figure 5A). 

### 3.6. A Causal Relationship between Intronization and Gene Expression Level Variation? 

Although highly expressed genes may preferentially accrue introns, the intronization process does not have to start in highly expressed genes. Coding sequences might take the intronization path in weakly expressed genes and contribute to boosting gene expression as their splicing levels increase. This hypothetical dynamic agrees with our observations (Figure 4) and with previous results showing that efficient splicing at the gene 5’ end can increase the transcription level [59,60,61,62,63]. 

**Figure 8:**
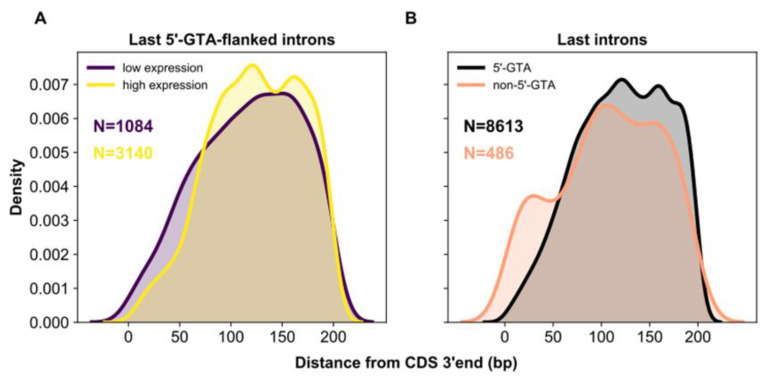
Distribution of last introns along the gene tail (arbitrary region: <200 bp). (**A**) In highly expressed genes (yellow), GTA-flanked introns less often reside in immediate vicinity to the gene 3′end than in lowly expressed genes (dark violet). (**B**) Last introns with a GTA 5′ splice site (black) tend to reside further away from the gene 3′end than those with a non-GTA 5′ splice site (light salmon).

Whereas intronization at the gene 5’ end may help boost gene expression level, it may have the opposite effect at the gene 3’ end [11,55]. At the gene 3’ end, the telescripting effects of optimal 5’ss-bound U1 snRNP can locally compromise the recruitment/operationalization of cleavage/polyadenylation factors (CPFs), ultimately dampening gene expression level [7,9,64]. In turn, CPFs may locally perturb the recruitment of U1 snRNP to 5’ss [11,55,65,66]. Drawing from these theoretical and empirical arguments, *Paramecium*’s last introns flanked by an optimal 5’ss (5’-GTA) are expected to reside farther apart from the gene 3’ end in highly expressed genes compared to weakly expressed genes. In addition, splicing should be generally disfavored at the gene 3’ end. Consistent with these expectations, 5’-GTA-flanked last introns reside, on average, farther from the CDS end of highly expressed genes compared to weakly expressed genes (e.g., within the terminal 200 bp: 129 bp vs 117 bp; Wilcoxon rank sum test, *p <* 2.2 × 10^−16^; Figure 8). Moreover, 5’-GTA-flanked introns reside farther from the CDS end compared to their suboptimal counterpart (i.e., non-5’-GTA-flanked introns), which is expected to recruit U1 less efficiently (e.g., within the terminal 200 bp: 125 bp vs. 110 bp; Wilcoxon rank sum test, *p* = 5.61 × 10^−15^; Figure 8). Finally, the gene 3’ end harbors the largest fractions of introns with non-zero retention levels and the highest average level of intron retention (Figure S1). Thus, not only intronization of coding sequences (Figure 4), but also splicing of annotated introns may be disfavored at the tail of highly expressed genes. Whether this excess of alternative intron retention at the 3’ end of *Paramecium* genes serves to tune gene expression as it does in mammals [67] requires further study.

### 3.7. Are Some Spliced Coding Sequences on Their Way to Becoming Exons?

Thus far, we have focused primarily on spliced GTA|TAG coding sequences. We have proposed that these sequences preferentially undergo intronization and that their efficient splicing at the gene 5’ end may increase gene expression levels. 

**Figure 9:**
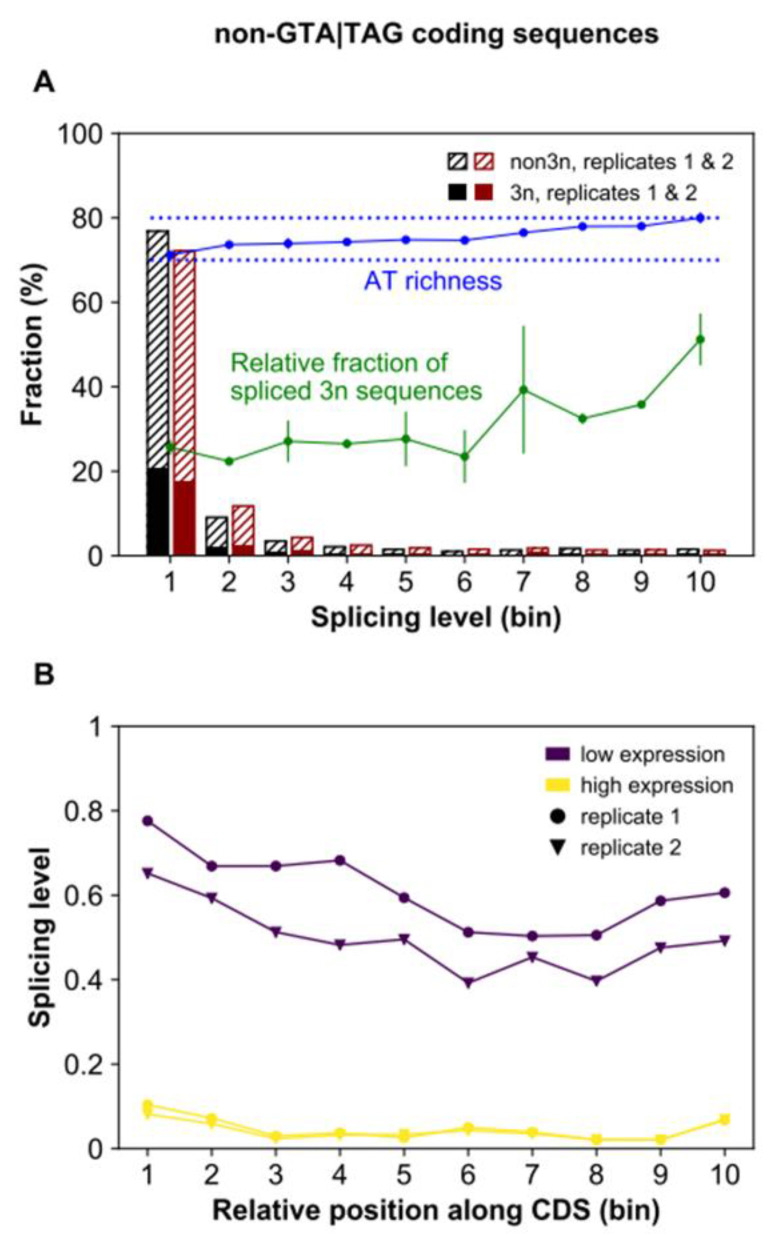
Splicing levels of non-GTA|TAG coding sequences. (**A**) Stacked bar heights represent the percentage of spliced sequences in each splicing level class (one of ten equal-width bins, e.g., bins 1 and 2 represent the splicing level intervals (0, 0.1] and (0.1, 0.2], respectively). Only loci covered by at least ten reads are considered. Solid and dashed bar parts (colored in black or red for the two surveyed replicates) correspond to 3*n* and non-3*n* spliced sequences, respectively. Further, the green and blue line plots show the relative fraction of 3*n* sequences and the AT content of the spliced sequences *per* splicing level class, respectively (mean +/- standard deviation of the two replicates in both cases). (**B**) Average splicing levels of spliced non-GTA|TAG coding sequences in each bin along the CDS were obtained for weakly (dark violet) and highly (yellow) expressed genes. Both the gene expression level and the relative position along the CDS vary with the splicing level of non-GTA|TAG coding sequences.

When we consider spliced non-GTA|TAG coding sequences, we find that they exhibit features that are strikingly similar to those of spliced GTA|TAG coding sequences. Specifically, most non-GTA|TAG coding sequences show low-level splicing (<10%) (Figure 9), whereas the fraction of 3*n* sequences increases with increasing splicing level (Rep1, Rep2, Pearson’s *r* > 0.72, *p* < 0.02; Figure 9), and the splicing level scales positively with AT content (Rep1, Rep2, Pearson’s *r* > 0.94, *p* < 3.86 × 10^−5^; Figure 9). Furthermore, their positional distribution varies with gene expression level in virtually the same way as spliced GTA|TAG coding sequences (Figure 9). The similarities between spliced GTA|TAG and non-GTA|TAG coding sequences raise an important question: why do non-GTA|TAG coding sequences not show signs of purifying selection as the GTA|TAG counterpart, but are instead associated with positive DSA estimates (Figure 1)? 

We speculate that spliced non-GTA|TAG coding sequences may undergo the opposite evolutionary path of exonization. In other words, at least some spliced non-GTA|TAG coding sequences may be former intronic sequences possibly on their way to becoming full-fledged coding exons. In line with this possibility, previous studies report that young (i.e., minor-form) exons tend to be unusually short, 3*n* in size when located within the coding region [68] and have weaker splice sites than constitutively spliced exons [69]. If so, then exonization, in contrast to intronization, may contribute to decreasing splicing efficiency (by increasing inter-intron distance, Figure 5), and reducing gene expression levels by preferentially subtracting the expression-enhancing effects of cap-proximal introns. Consistent with this hypothetical scenario, spliced non-GTA|TAG coding sequences are over-represented in highly expressed genes (73% vs. 54%, respectively; proportion test, *p <* 2.2 × 10^−16^, Figure 4A,B) and tend to reside at these genes’ 5’ end (high expression: χ^2^ = 100.27, df = 9, *p* < 2.2 × 10^−16^, bin1/bin10 = 2.6; low expression: χ^2^ = 19.56, df = 9, *p* = 0.021, bin1/bin10 = 1.7). 

### 3.8. Intronization Promotes Exon Erosion 

**Figure 10:**
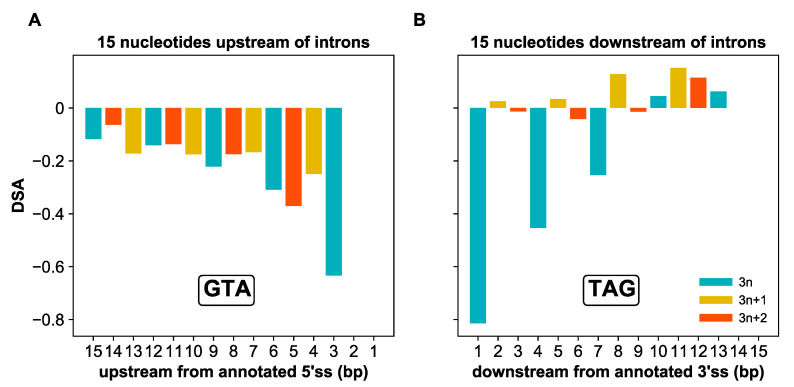
Strong cryptic splice signals are counter-selected in the vicinity of annotated introns. (**A**) DNA strand asymmetry (DSA) scores of the GTA trinucleotide in the 15 nucleotides (nt) upstream of the annotated 5′ splice site (5′ss). DSA values are most negative directly upstream of the canonical 5′ss and increase with increasing distance, suggesting that the selective pressure is highest directly adjacent to the annotated 5′ss. (**B**) DSA scores of the TAG trinucleotide in the 15 nt downstream of the annotated 3′ splice site (3′ss). Similar to 5′ss, negative selection is strongest in the immediate vicinity of the annotated 3′ss. However, the signature of counter-selection against cryptic TAGs affects only ~10 nt downstream of the true 3′ss and is almost exclusively limited to distances that would enlarge the intron by 3*n* nucleotides.

Finally, intronization does not involve only the emergence of whole new introns. Rather, it can also reflect the incorporation of exonic tracts into existing introns [20]. To gain insights into this putative process of exon erosion, we first examined the degree of DNA strand asymmetry of the GTA trinucleotide within 15nt upstream from canonical 5’ss. We find that this trinucleotide is counter-selected, particularly near the canonical 5’ss (Figure 10). We next examined the distribution of the TAG trinucleotide along the downstream exon tract. We also detected a signature of negative selection immediately downstream from canonical 3’ss (Figure 10). Unlike the GTA trinucleotide though, the signature of selection against TAG has a 3-nucleotide period and disappears entirely 10nt away from the 3’ss (Figure 10). The selective pressure against GTA and TAG trinucleotides around *Paramecium* introns suggests that intron size in this organism is unlikely to grow any larger. When it does, our findings suggest that introns are likely to capture exonic tracts downstream more than upstream from introns and to incorporate non-3*n* adjacent exonic tracts.

## 4. Discussion

The compartmentalization of intragenic sequences into exons and introns is entrenched in contemporary biology. For decades, introns have been viewed as the evolutionary byproduct of exogenous insertions [70,71] (but see [72,73,74,75]) and their gains (and losses) as binary events. Moreover, the effects of intron turnover on the evolution of gene properties have often been neglected (but see [19,20,76]) despite the established interactions between RNA splicing and other mRNA-associated processes [6]. 

Our findings suggest that there is evolutionary fluidity between coding exons and introns. The uncovered signatures of intronization provide strong, inevitably indirect, evidence that over evolutionary time, *Paramecium* introns emerge genome-wide from coding sequences due to splicing errors and reduced selective constraints. They suggest that intron gain is a gradual process, which involves a transient phase of alternative splicing. This process is preferentially completed at the 5’ end of highly expressed genes, when spliced 3*n* coding sequences acquire premature termination codons, and where spatial constraints and/or splicing-antagonistic interactions are reduced relative to downstream regions. Importantly, decreased selective constraints may drive not only the origin of introns but also their lengthening at the expense of flanking exons—provided that the spliceosome range of action is not inherently constrained as it might be in *Paramecium*. 

The uncovered signatures of intronization provide several new insights on old questions or common assumptions. First, because coding sequences are preferentially spliced in weakly expressed genes, but they may preferentially achieve a full-fledged intron status in highly expressed genes, it is possible that episodes of intronization enhance gene expression level. This possibility aligns with the expression-enhancing effect of optimally spliced introns at the gene 5’ end [59,60,61,62,63]. It also points toward a role of exon-to-intron (and intron-to-exon) sequence conversion in adaptive evolution. 

Second, the suggestion that introns grow larger at the expense of flanking exons provides a simple mechanistic explanation for a common observation: across the eukaryotic tree, species with large introns tend to have small exons, and vice versa [77]. 

Third, splice site recognition has been traditionally viewed as a process that encompasses either exons or introns (the exon- and intron-definition models) [78,79,80]. An evolutionary fluidity between exons and introns is barely compatible with these models, but favors spliceosomal dynamics that are independent from an underlying sequence “category” [19,81]. 

Fourth, the genome-wide signature of selection against GTA and TAG trinucleotides strongly suggests that a fraction of synonymous sites in *Paramecium* are not evolving neutrally, with consequent inflating effects on widespread statistical tests for the inference of positive selection, such as *dN/dS* [82]. 

Fifth, and lastly, our observations support an intimate link between intron gain, alternative splicing, and the power of natural selection/genetic drift [19]. They predict that alternative splicing and intron gain are more frequent when the power of selection is reduced relative to the power of random genetic drift [19]. This implies that intron gains may be viewed as mildly deleterious innovations [83], and that organisms with relatively low intron density (most often single-celled species [84]) may evolve under higher levels of selection intensity compared to multicellular organisms such as humans (where exons have reached minimal size in many genes) [85,86]. Following this rationale, inefficient/relaxed purifying selection may have driven intron accumulation in the intron-rich last eukaryotic common ancestor [87]. Another prediction is that recent sequence insertions in a genome (e.g., transposon insertions) may be the preferential target of the splicing machinery as the cryptic splice sites that they may contain have not yet been counter-selected, which is in line with recent findings [21]. 

Overall, our findings suggest that the gene architecture of *Paramecium* has undergone considerable changes during evolutionary time. More in general, genomic sequences that experience relaxed selective constraints (e.g., upon exposure to an environmental change) and/or have not yet experienced negative selection (e.g., evolutionarily recent transposon insertions, horizontally transferred sequences) are predicted to be preferential targets of the splicing machinery and thus are more likely to be coupled with exon-to-intron sequence conversion. The current gene architecture can be thought of as a snapshot of an ongoing evolutionary process and much of the alternative splicing as a manifestation of this process.

## Figures and Tables

**Table 1:** Frequency (in percentage) of *Paramecium* introns with putatively optimal signals, at both splice sites simultaneously (GTA-TAG), at the 5’ss (GTA-nAG), or 3’ss (GTn-TAG) at different levels of gene expression (quartiles based on the vegetative expression level as in [48]).

Splice Sites	Expression Level			
	*Low*	*Medium Low*	*Medium High*	*High*
GTA-TAG	65.5	66.9	69.3	72.6
GTA-nAG	91.8	94.0	94.7	94.9
GTn-TAG	70.5	71.3	73.4	76.5

## Data Availability

The data presented in this study are openly available in Zenodo at https://zenodo.org/record/6141833.

## Supplementary Materials

The following supporting information can be downloaded at: https://www.mdpi.com/article/10.3390/microorganisms10101901/s1, Figure S1: Fraction of introns with non-zero retention levels by position and splice-site type.
