# Supplementary material for: Intronization Signatures in Coding Exons Reveal the Evolutionary Fluidity of Eukaryotic Gene Architecture
Source: Microorganisms. 2022 Sep 25;10(10):1901. doi: 10.3390/microorganisms10101901 (PMC9612004; doi:10.3390/microorganisms10101901)
Supplement: Supplementary file 1 [file microorganisms-10-01901-s001.zip › microorganisms-1895197-supplementary.pdf]

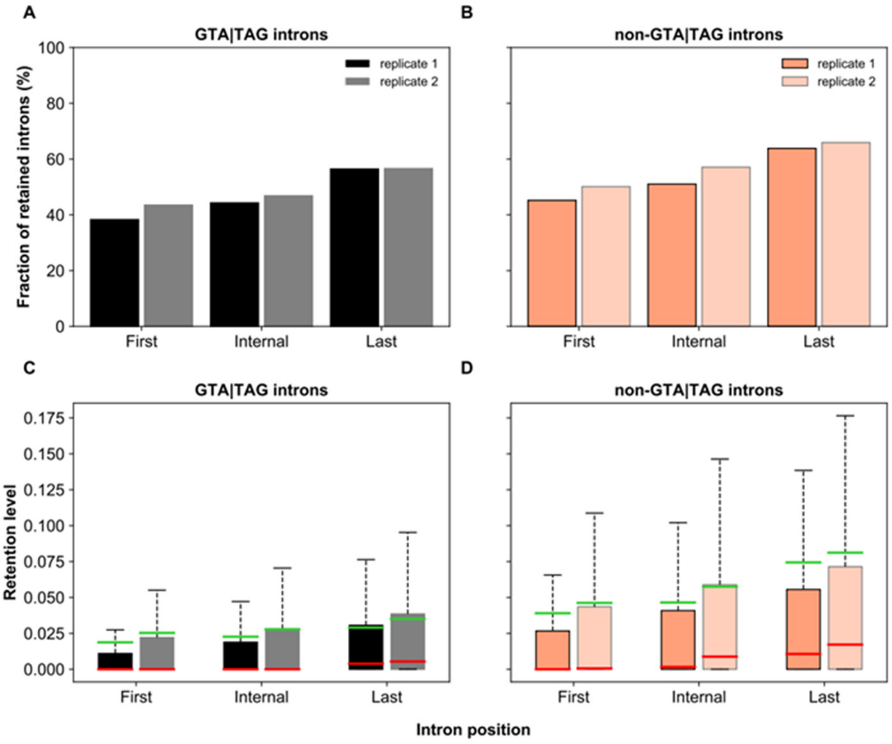

Figure S1. Bar plots showing the fraction of introns with non-zero retention levels in different positional classes (first, internal, and last in genes with >2 introns) for GTA|TAG (**A**) and non-GTA|TAG (**B**) introns. In both surveyed replicates (coloring according to legend), GTA|TAG introns show lower fractions of retained introns compared to non-GTA|TAG introns. The fraction of retained introns increases from first to last introns regardless of the splicing signal strength. Boxplots showing the retention levels in first, internal, and last introns (genes with >2 introns) for introns with GTA|TAG (**C**) and non-GTA|TAG (**D**) signals. Mean and median retention levels are indicated by green and red horizontal lines, respectively, and outliers are omitted for clarity. Average retention levels are lower for GTA|TAG introns than for non-GTA|TAG introns. Irrespective of the splicing signal strength, the first introns show the lowest and last introns the highest average retention levels, respectively.
